# Nutrient Deficiencies and Potential Alteration in Plasma Levels of Naturally Acquired Malaria-Specific Antibody Responses in Tanzanian Children

**DOI:** 10.3389/fnut.2022.872710

**Published:** 2022-06-17

**Authors:** Erasto V. Mbugi, Gerco den Hartog, Jacobien Veenemans, Jaffu O. Chilongola, Hans Verhoef, Huub F. J. Savelkoul

**Affiliations:** ^1^Cell Biology and Immunology Group, Department of Animal Sciences, Wageningen University, Wageningen, Netherlands; ^2^Department of Medical Biochemistry and Molecular Biology, Kilimanjaro Christian Medical University College, Moshi, Tanzania; ^3^Department of Biochemistry, Muhimbili University of Health and Allied Sciences, Dar es Salaam, Tanzania; ^4^Nutrition and Public Health Intervention Research Unit, London School of Hygiene and Tropical Medicine, London, United Kingdom

**Keywords:** nutrient deficiencies, zinc, magnesium, malaria antibodies, IgG subclasses

## Abstract

Immunoglobulin G (IgG) subclasses have been suggested to confer naturally acquired immunity to Plasmodium falciparum malaria. Cytophilic IgG1 and IgG3 with their potential for opsonization, phagocytosis, and antibody-dependent cellular inhibition in association with monocytes have been suggested to have a critical role in malaria. The potential for production of antibodies is influenced by micronutrient status. This study aimed at exploring the effect of micronutrients, particularly zinc status, on the profiles of IgG subclasses in 304 Tanzanian children aged ≤ 5 years. An enzyme-linked immunosorbent assay was performed using whole asexual blood stage malaria antigens to determine plasma malaria-specific antibody titers. This baseline cross-sectional study was done from 2005 – 2010 prior to the larger randomized control trial of the Micronutrient and Child Health (MACH) Study. Plasma concentrations of zinc and magnesium were measured by inductively coupled plasma atomic emission spectrometry and results correlated with plasma IgG subclass levels. The findings reveal zinc deficiency to possibly influence the production of IgM, total IgG, and several IgG subclasses in a malaria status-dependent manner. Among IgG subclasses, IgG3 and partly IgG2 displayed a remarkable association with zinc deficiency, particularly IgG3 which was predominant in children with malaria. Nevertheless, zinc, magnesium, and malaria status did not influence the association between IgG3 and IgG4. The study leads to the conclusion that, under conditions of micronutrient deficiency and malaria status, an imbalance in IgG subclass production may occur leading to predominantly higher levels of IgG3 and IgG2 that may not confer sufficient protection from infection. The profile of both cytophilic and non-cytophilic IgG subclasses has been shown to be variably influenced by zinc status; the effects vary with age at least in under-fives. These results provide insight for inclusion of micronutrients, particularly precise amounts of zinc, in future malaria interventional programs in endemic areas.

## Introduction

The humoral immune response is mediated by naturally acquired antibodies against *Plasmodium falciparum* blood-stage surface antigens and is vital in limiting parasite multiplication and the conferral of protection to clinical malaria. In malaria endemic areas, the development of naturally acquired immunity to severe disease takes place in children at an age range of 1 – 5 years (1). This ‘antiparasite’ immunity is not absolute and is acquired through repeated exposure to *Plasmodium falciparum* (2) starting early in life (3), and may be hindered by exposure-reduction interventions (4). Many parasite antigens are known to occur at different stages of the parasite in the human host with the body generating antibodies against the prevailing antigenic proteins. Protective immunity to falciparum malaria, however, has particularly been associated with cytophilic antibodies of immunoglobulin G (IgG) subclasses (1, 2). Ferrante and Rzepczyk (2) pointed out the switch from immunoglobulin M (IgM) on B cells to different isotypes and different IgG subclasses (IgG1, IgG2, IgG3, and IgG4) upon encounters with malaria antigens. These antigens can differentially modulate immunoglobulin heavy-chain switching through induction of different cytokine secretory patterns by CD4^+^ T helper (Th) cells (2, 5). The differential release of these cytokines (type I or type II) is influenced by nutritional status, particularly zinc (6, 7). Studies by Tongren et al. (8) reported the regulation of immunoglobulin class switching in murine malaria to be epitope-specific and that in human malaria, IgG1/IgG3 class switching is independently regulated by the nature of the antigen, cumulative exposure to the antigen, and maturity of the immune system (9).

Several studies have highlighted the importance of naturally acquired antibody-mediated immunity through IgG subclasses to be crucial in limiting clinical malaria (2, 10–18), although these studies have targeted different parasite surface antigens. The levels of subclasses of IgG, in particular the proportion of cytophilic (IgG1 and IgG3) to non-cytophilic (IgG2 and IgG4), have been hypothesized to be more significant than the overall levels of antibodies in providing protection to the development of severe disease (1, 19–21). In addition, the fine specificity of antibodies toward specific antigenic epitopes on *P. falciparum* antigens is thought to be critical for the generation of an effective immune response (20, 21).

In early life, acquisition of natural humoral immunity to *P. falciparum* is key in endemic areas and is said to be influenced collectively by clinical, environmental, and host factors (22). The immunological balance between host and parasite in malaria is important in determining the fate of infection (23) and may largely be influenced by nutritional status. Poor nutrition in children may interfere with the development and function of the immune system. The immune regulatory mechanisms become impaired both quantitatively and qualitatively due to altered immune cell populations (7, 19, 24–26) as a result the whole immune response pathway from innate to adaptive antibody response including cellular responses is said to be affected (7, 27–38). In malaria endemic areas, the impact of the disease may even be aggravated. Most children become vulnerable to infections at weaning when the passive immunity acquired from prenatal maternal antibodies and breast milk wanes. During this transition period, the nutritional support is subject to instability and inclined to depletion. It is likely that poor nutrition interferes with the immune response to malaria in African settings where both situations prevail. Zinc ions, for example, have been hypothesized to be involved in regulating signal transduction in various immune cells playing a second messenger role (39). The philosophy underlying the immune dysfunction-malnutrition connection informs the cause-consequence idea such that each may be a cause or consequence of the other (40). Several other reviews and studies (31, 35–38, 41–47) have explored the association between micronutrients, particularly zinc deficiencies, with infections but only a few have specifically focused on the association with malaria (41, 44). Even those studies that have extensively investigated the factors associated with a protective role of IgG subclasses have been limited to individual effects of age, exposure, transmission intensity, ethnicity, geographical location of the parasite, seasonality on the dynamics (13, 42), and fine specificity (13) of antibody-mediated protection to malaria. The fact that, in some children, the B-cell response undergoes isotype switching to a more cytophilic antibody response to malaria early in life and those children remain protected from disease attacks for longer time periods than other children (9, 43), even when exposure is limited by seasonality (2, 47), is indicative of the contribution of other factors like nutritional status in strengthening the effectiveness of antibody response to malaria. Franca et al. (48) propose the challenge of malnutrition to immunity and infection and suggest proper medical-nutritional management to prevent adverse effects of malaria infection (49) despite existing variable debates (50). Some studies have principally indicated, despite evidence of malnutrition, that children develop significant antibody-mediated responses to common pathogens (51), signifying immune response build up with malaria periodic exposure throughout life in endemic areas (52).

The study hypothesized that zinc and other micronutrients deficiencies induce relative alterations in the plasma profile of naturally acquired *Plasmodium falciparum*-specific antibody responses that may influence the induction of a protective immunity to manifestation of clinical disease.

## Materials and Methods

### Study Area and Population

The cross-sectional study was conducted in the period of May 2005 – July 2006 in a lowland area around Segera village (S 05° 19.447', E 38° 33.249'), Handeni District of Tanga, north-eastern Tanzania. Malaria is highly endemic in this area, with virtually all infections being due to *P. falciparum*. Since then, no similar studies have been conducted in Tanzania or in this highly malaria endemic African area. The residents in the study population comprise mostly poor farmer families growing maize and cassava for subsistence use. At the time of this study, only one health center in Segera was available to serve all of the surrounding area. The study was approved by both Ethics Review Committees in the Netherlands and Tanzania (for Tanzania ethics review bodies, the reference numbers for Kilimanjaro Christian Medical Center (KCMC) and National Ethics Review Committee were 094 and NIMR/HQ/R.8a/VolIX/540, respectively). Informed consent was obtained from community leaders and local government officials, and from parents or guardians.

### Sampling Methods and Eligibility Criteria

A census list was made with all resident children aged 6−72 months in the study area. Using this list, 16 children were randomly selected from each of 19 communities, resulting in a total of 304 children. Children were eligible when they had no fever, and showed no signs of other severe disease or severe malnutrition (weight-for-height z-score below−3 SD). Further details are provided elsewhere (53).

### Field Procedures

All children were examined by clinical officers, who also measured axillary temperature by electronic thermometer. Information on the state of health such as malaria, fever, sickness, and reports on fever in the past 14 days was diligently recorded. A project phlebotomist collected fasting venous blood samples (6 mL) in containers (vacutainer tubes) suitable for mineral element analysis with sodium heparin as the anticoagulant (Becton-Dickinson, Franklin Lakes, NJ). Immediately upon collection, the cap was sprayed with ethanol and allowed to dry; approximately 1.3 mL of blood was then drawn in a sterile manner from the collected blood into a different tube for different measurements including peripheral blood mononuclear cells (PBMCs), plasma for ELISA, parasite count, etc. Children were treated for common childhood infections and anemia according to the guidelines of the Tanzanian Ministry of Health.

After arrival at the laboratory in Moshi, blood samples were immediately centrifuged (300 *g*) at ambient temperatures for 10 min and plasma obtained from the centrifuged blood (1.2 mL) was stored in liquid nitrogen, and subsequently transported on dry ice to the Netherlands to measure additional biochemical indicators of micronutrient status and inflammation (54, 55). Further processing that involved PBMC isolation-yielded plasma was used for ELISA whose results are reported in this paper.

### Parasite Enzyme-Linked Immunosorbent Assays (ELISA)

Different ELISAs testing antibodies against various specific parasite antigens are described elsewhere (1, 12, 14, 15, 56–58). The project developed its own direct ELISA test protocol that was used to determine the plasma concentration of isotype-specific antibodies responding to *Plasmodium falciparum* asexual blood stages. Parasite extract (asexual blood stage antigens) was used to coat the plates, and these were covered and incubated overnight at 4^0^C. The parasite extract was obtained as a kind gift from Professor Robert Sauerwein from the Medical Parasitology Laboratory, Radboud University Medical Center Nijmegen, the Netherlands. The plates were blocked with 150 μL of 1% w/v bovine serum albumin, (BSA) (grade V, Sigma P4417, St Louis MO, USA) in phosphate-buffered saline (PBS). After blocking, the plates were washed three times with an extensive volume of 0.05% Tween 20 (v/v) (Sigma-Aldrich, Missouri, USA) in PBS. Plasma samples were diluted five (IgG total, IgG1, IgM) or 20 (IgG2, IgG3, IgG4) times in 0.2% w/v BSA and 0.05% Tween 20 in PBS. Plasma samples were added to the wells at 50 μL and malaria-positive and negative samples were added as controls. The positive control consisted of a pooled sample of 25 highly immune Tanzanian individuals. Addition of plasma to the wells was followed by incubation under cover for 3 h at room temperature. After incubation, the plates were washed three times in 0.05% Tween 20 in an extensive volume. Thereafter, 50 μL of horseradish peroxidise (HRP) conjugated sheep anti human antibody (The Binding Site, Birmingham, UK) was added and incubated at room temperature in a shaker in the dark for 1.5 h. The following concentrations were used: 1 μg/ml for IgG total (AP003) and IgG1 (AP006) and 2 μg/ml for IgM (AP012), IgG2 (AP007), IgG3 (AP008), and IgG4 (AP009). Plates were emptied and washed six times in 0.05% Tween 20 and finally 100 μL of substrate (2.2'-azino-di[3-ethylbenzthi-azoline sulfonate (59)]) and ABTS (Roche Diagnostics, Mannheim, Germany) was added and incubated in the dark for color development. A plate reader (Anthos Photometer 2020, Anthos Labtec Instruments GmbH, Wals, Austria) was used to measure color development at 405 nm.

### Determination of Plasma Concentrations of Mineral Elements

Plasma samples were diluted 20 times in milliQ (60) and concentrations of zinc and magnesium were measured by inductively coupled plasma atomic emission spectrometry (ICP-AES) (Vista Axial, Varian, Australia). To determine variability in outcomes, measurements were replicated five times. With mean values set at 100%, measurements varied between 97 and 102% for zinc and 99% and 102% for magnesium. Cut off values were set for zinc (plasma zinc concentration <9.9 μmol/L), magnesium (plasma magnesium concentration <750 μmol/L), and iron deficiency anemia (iron deficiency, ferritin concentration <12 μg/L accompanied by anemia, hemoglobin concentration <110 g/L). Mineral determination included plasma copper but no evidence was found for copper deficiency as assessed by plasma copper concentrations < 7.1 μmol/L (unpublished data). Therefore, only the results for zinc and magnesium and its association with IgG subclass levels in respect to malaria infection are reported in this paper. Iron deficiency anemia was defined as a ferritin concentration <12 μg/L accompanied by anemia, hemoglobin concentration <110 g/L.

### Statistical Analysis

The data were entered and analyzed using SPSS for Windows (version 15.0. SPSS Inc., Chicago, IL, USA). The previously defined zinc and magnesium deficiencies (55) were used in this analysis. Relative titers were calculated using the control sample which was added to each plate. For these calculations, a standard curve was made. Antibody titer values were log-transformed to obtain normally distributed variables. A linear regression model was used to calculate the unit change in antibody levels in zinc-replete and deficient individuals. Linear regression analysis was also used to assess whether different IgG subclass associations were influenced by conditions of zinc, magnesium, iron deficiency anemia, and malaria status. Average antibody titers were determined by comparison of means from which the standard error of the means was used to calculate confidence intervals and the absolute values were obtained by exponential transformation of previously log-transformed values using Microsoft Excel. The difference in antibody titers in relation to zinc status among groups was based on ‘healthy’ children (with neither condition) and unit change estimated for asymptomatic malaria (from malaria dipstick results), children reported sick with malaria infection, children reported sick with malaria infection and a history of fever in the past 24 h, and those reported sick without malaria infection. The group differences were accounted for by the use of confidence intervals (95% CI and *p-*value < 0.05). A generalized linear model (GLM) was utilized to determine whether the effect of zinc deficiency on plasma IgG subclasses was age-dependent. Lots were used as scaling weight variables to account for the diversity in malaria prevalence among lots. The analysis compared the differences among age groups in *P. falciparum* (schizont asexual blood stage parasites)-specific antibodies plasma reactivity. Three age groups were established: 0.5 – 1.5 years, 1.5 – 3 years, and 3 – 5 years.

## Results

### General Characteristics of the Study Population

This study was carried out in a population of children consisting of 135 boys and 169 girls with similar age distributions. Details of population characteristics and other relevant information are revealed in previous similar reports (53–55).

### Total IgM and IgG Plasma Levels Relative to Zinc Status

Initially the possible difference in levels of IgM and total IgG class (Tables 1, 2) relative to zinc status was evaluated. The relative antibody levels in the zinc-replete group were moderately higher compared to the zinc-deficient group. IgM levels were significantly increased relative to the reference group (healthy children) in children with asymptomatic malaria and those reported sick with malarial infection and reported fever in the past 24 h in the zinc-replete group. Significantly higher levels of IgM were recorded in the zinc-deficient group for asymptomatic children, sick with malaria, and those who were reported sick with malaria and a report of fever in the past 24 h (Table 1). The results in total IgG levels followed similar trends to the levels of IgM for the zinc-replete as well as the zinc-deficient group (Table 2). In both cases, the increase in IgM and total IgG levels were statistically not significant in children reported sick but malaria-negative.

**Table 1:** Variation in plasma total IgM levels in different health situations associated with malarial infections in zinc–deficient subjects.

**Healthy status**	**IgM**							
	**Zinc replete**				**Zinc deficient**			
	**Mean level (n)**	**Difference**	**95% CI**	***P*–value**	**Mean level (n)**	**Unit change**	**95% CI**	***P*–value**
Healthy children	118.4 (47)				112.0 (46)			
Asymptomatic malarial infection	164.0 (38)	38%	16% to 65%	0.000	161.2 (41)	44%	23% to 69%	0.000
Sick with malarial infection	181.7 (09)	53%	25% to 89%	0.000	171.8 (10)	53%	17% to 100%	0.002
Sick with malarial infection and 24 h history of fever	134.5 (22)	14%	−15% to 52%	0.389	175.6 (09)	57%	21% to 103%	0.001
Sick without malarial infection	109.7 (30)	−7%	−23% to 12%	0.419	106.1 (32)	−5%	−20% to 12%	0.529

*Statistical test, Comparison of Means*.

**Table 2:** Variation in plasma IgGT levels in different health situations associated with malarial infection in zinc-replete subjects.

**Health status**	**IgGT**							
	**Zinc replete**				**Zinc deficient**			
	**Mean level (n)**	**Unit change**	**95% CI**	***p*-value**	**Mean level (n)**	**Unit change**	**95% CI**	***p*-value**
Healthy children	80.1 (47)				86.8 (46)			
Asymptomatic malarial infection	112.8 (38)	41%	13% to 75%	0.002	119.5 (41)	38%	14% to 66%	0.001
Sick with malarial infection	122.5 (09)	53%	18% to 98%	0.001	125.7 (10)	45%	6% to 98%	0.021
Sick with malarial infection and 24 h history of fever	110.6 (22)	38%	−4% to 98%	0.081	123.8 (09)	43%	6% to 93%	0.021
Sick without malarial infection	68.3 (30)	−15%	−32% to 8%	0.177	81.5 (32)	−6%	−23% to 15%	0.534

*Statistical test, Comparison of Means*.

### Difference in Plasma Levels of IgG Subclasses

**Figure 1:**
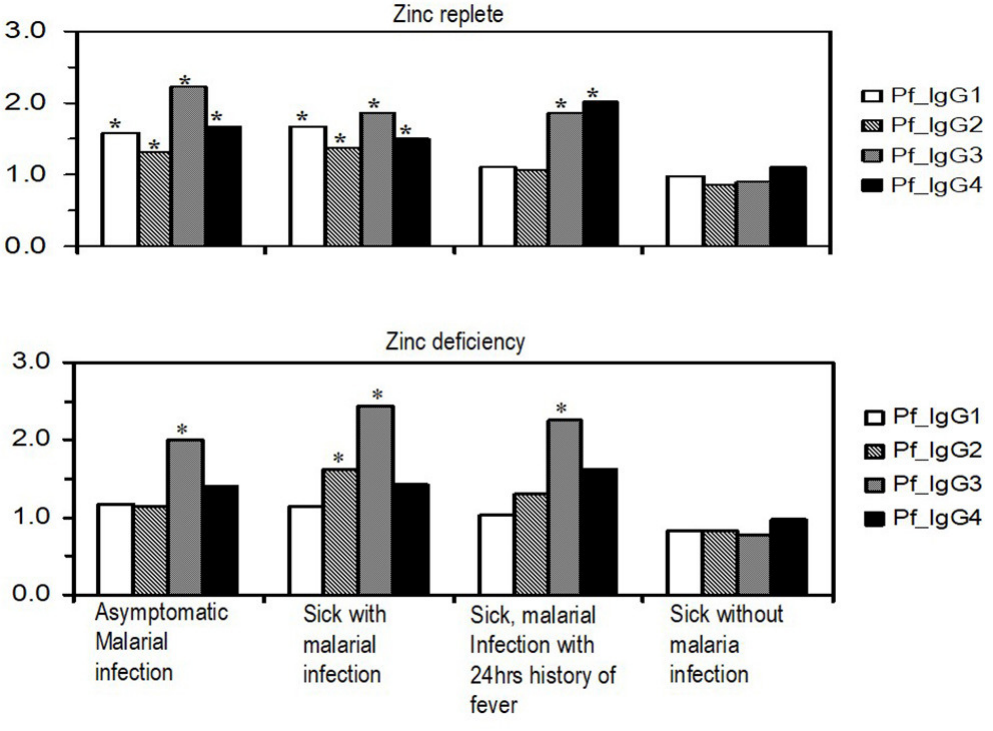
Variations in profile of relative plasma concentrations of malaria parasite-specific IgG subclasses in different malaria situations with and without associated clinical features in zinc-replete and zinc-deficient children. Values on the y-axis are log-transformed values of antibody titers as detected in plasma. Bars with asterisks (*) indicate significance at *p* ≤ 0.05.

Evaluation of the general plasma changes in IgG subclasses in different malaria situations is shown in Figure 1. There was a significant change in antibody titers for all IgG subclasses (*P* < 0.05) in zinc-replete children with asymptomatic malaria and those who were sick and had malaria infection. In these subjects, the changes in IgG3 and IgG4 levels were almost the same with IgG4 being slightly higher than IgG3 in children reported sick with malaria and those who were reported sick with malaria infection and a report of fever in the previous 24 h. With zinc deficiency (Figure 1), only the changes in IgG3 levels were significant for asymptomatic malaria, sick with malaria infection, and in sick children with malaria who reported complaints of fever in the past 24 h (*P* < 0.05). On the other hand, IgG2 predominantly showed increased levels in children reported sick with malarial infection (*P* < 0.05). There was a marked difference with respect to zinc status in which only IgG3 and partly IgG2 predominated in zinc deficiency which may imply that these IgG subclasses remain as critical weapons to fight against malaria in nutrient deficiencies. The insignificant change in IgG subclasses in children reported sick without malaria infection strengthens the notion that the antibodies studied are specific for malaria antigens.

### Comparison of Levels of Plasma IgG Subclasses With Respect to Zinc Status

**Figure 2:**
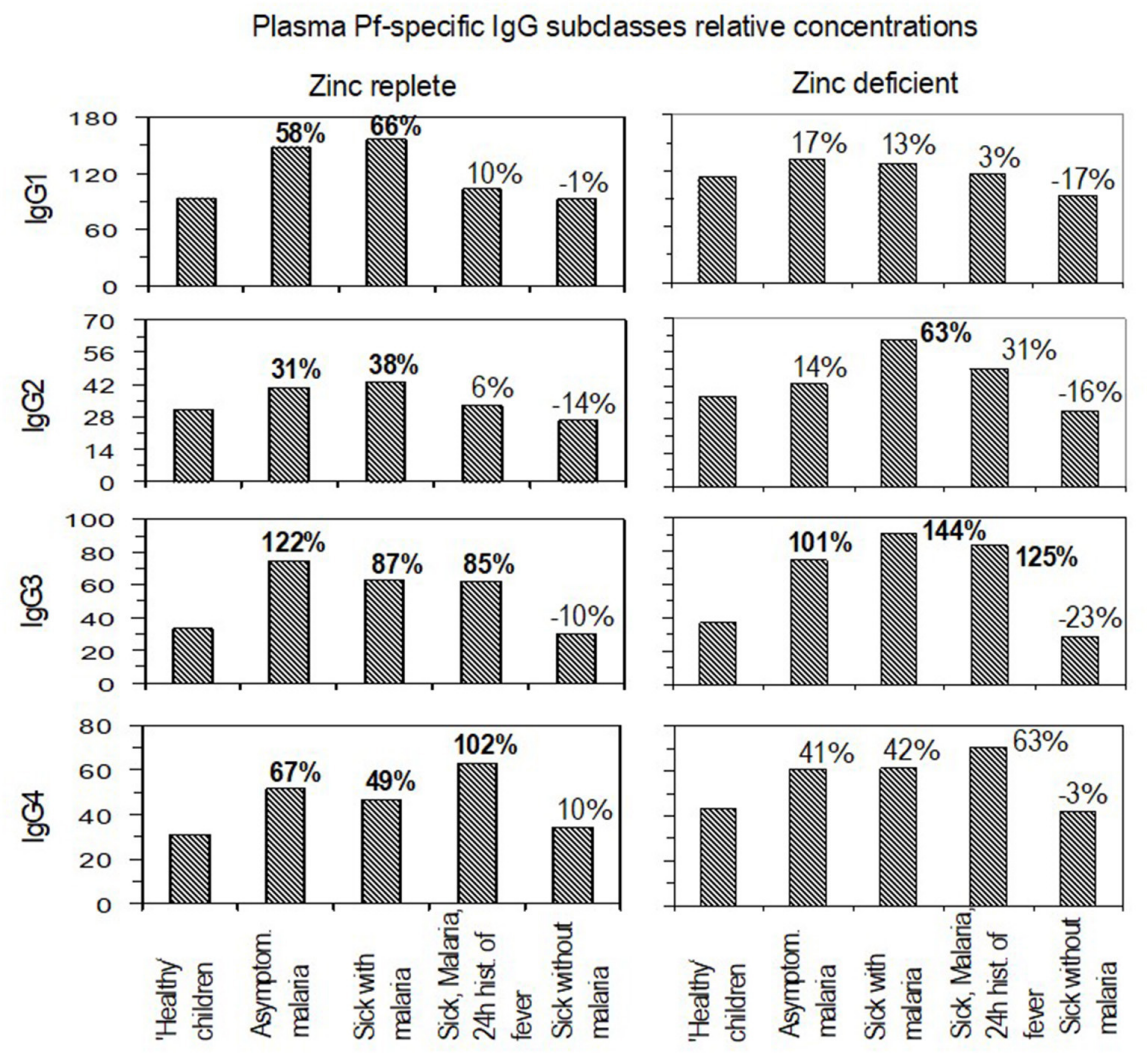
Relative plasma levels and unit change under different malaria and zinc statuses. For each immunoglobulin G subclass, the panels compare the levels in zinc-replete and zinc-deficient situations at different states of malaria infections. Percentages indicate paired group unit change differences in the relative plasma levels of antibodies. Bolded values: significant at *p* ≤ 0.05.

The levels of *Plasmodium falciparum*-specific IgG subclasses in plasma were assessed to determine whether there were differences within individual IgG subclasses with respect to zinc status (Figure 2). With ‘healthy children’ set as the reference group, the levels of IgG1 were significantly higher in asymptomatic malaria (*P* = 0.007; 95% CI, 13 to 120%) and in children who were reported sick and found to be malaria-positive by dipstick (*P* = 0.011; 95% CI, 13 to 146%). There was no significantly high levels of IgG1 in the zinc-deficient group. Increase in IgG2 levels followed a similar trend to IgG1 in the zinc-replete group, but in the zinc-deficient group, the IgG2 levels were significantly higher in the children reported sick and found positive for malaria (*P* = 0.015; 95% CI, 10 to 141%). Levels of IgG3 were significantly higher in both zinc-replete and zinc-deficient groups (*P* < 0.05) relative to healthy individuals, except in children reported sick without malaria (Figure 2). The levels of IgG3 in the zinc-deficient group reported sick children with malaria (*P* = 0.004; 95% CI, 30 to 289%) and those reported sick with malaria and a history of fever in the past 24 h (P = 0.002; 95% CI, 38 to 333%) were significantly and relatively higher than their counterparts in the zinc sufficient group. With the exception of children who were reported sick without malaria, IgG4 levels were higher in the zinc-replete group and the higher levels of IgG4 in the zinc-deficient group in all cases were statistically insignificant (Figure 2).

### Association Between IgG3 and IgG4 Subclasses Under Different Conditions of Micronutrients and Malaria Status

**Figure 3:**
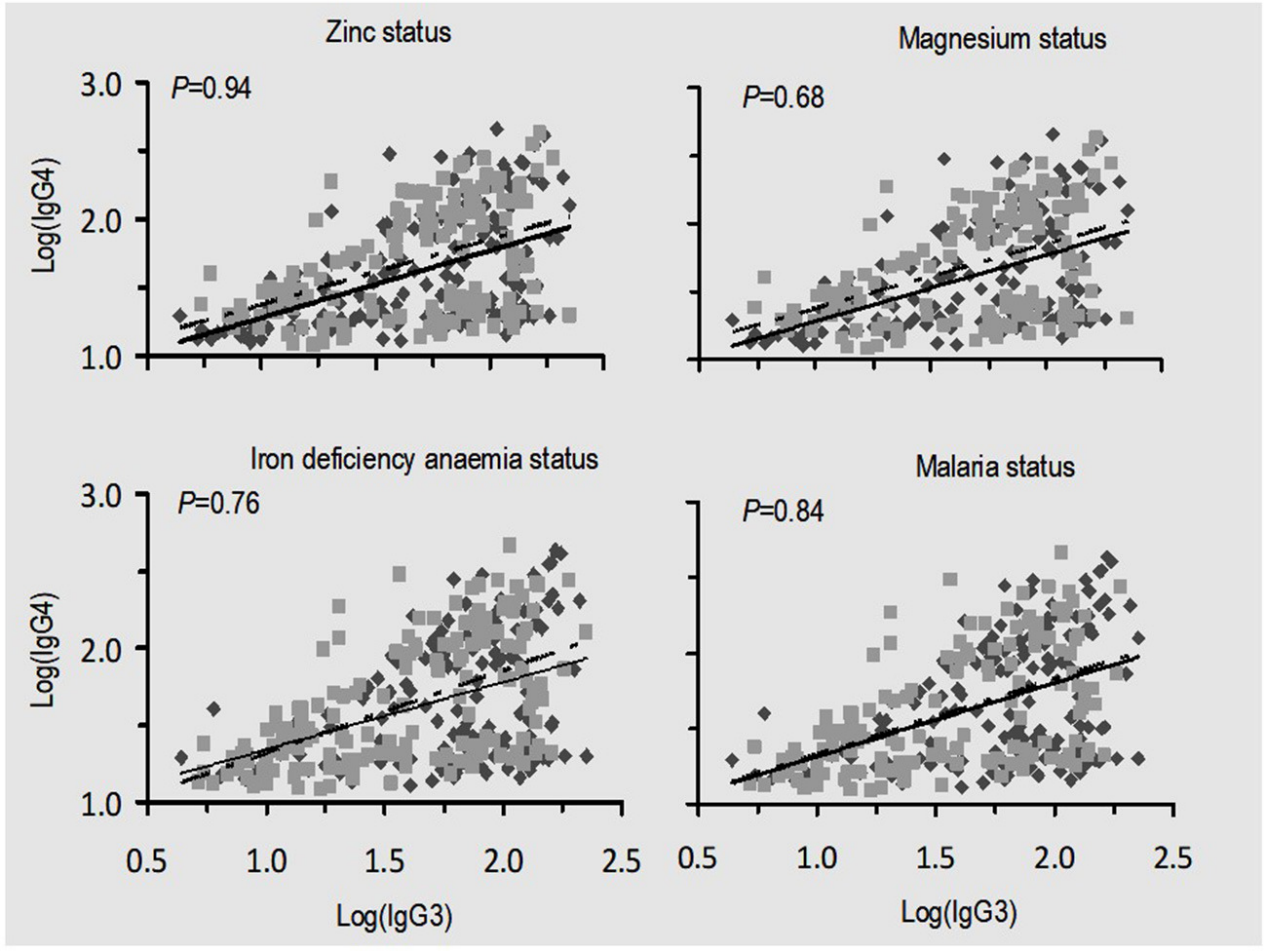
Relationship between malaria-specific plasma IgG3 and IgG4 under different situations of nutrition and malaria status. There were no significant differences between slopes in all four conditions being compared as indicated by *p*-values. Scatter spots (black): zinc and magnesium replete, absence of iron deficiency anemia, and absence of malaria infection; scatter spots (gray): zinc, magnesium deficiency, and iron deficiency anemia and malaria infection. The solid black and dashed lines are their corresponding regression lines.

Since the levels of IgG subclasses, particularly IgG3 and IgG4, showed greater potential for variation in levels with respect to zinc status, a linear regression analysis was performed to determine whether these two immunoglobulin subclasses were associated under different conditions of micronutrient status (Figure 3). The association between IgG3 and IgG4 was linear but the higher *P-*values reflected that the slopes of the regression lines in the four panels (Figure 3) were not different. These results provide no evidence that the associations between IgG3 and IgG4 are influenced by deficiencies in zinc, magnesium, iron deficiency anemia, or malaria status.

### Effects of Zinc Deficiency on IgM, Total IgG, and Malaria-Specific IgG Subclasses With Age Group

Using a generalized linear model, we assessed whether the influence of zinc deficiency on the change of IgM, IgG total, and IgG subclass levels varied with age (Table 3). Results showed that zinc deficiency significantly influenced the change in antibody levels with variable conditions of malaria status and age groups. The reduction in levels of IgM and IgG3 had more of an impact on older children (3 – 5 years) as compared to other age groups. On the other hand, the impact of zinc deficiency on IgG1 and IgG4 was significant in the younger age (0.5 – 1.5 years) group with an insignificant impact on the medium (1.5 – 3 years) and the older age groups. The effect of zinc deficiency on IgG2 in children reported sick without malaria and on IgG3 in children reported sick with malaria was borderline significant (Table 3).

**Table 3:** Variation in levels of IgM, IgG total, and IgG subclasses due to age in zinc-deficient children compared to the healthy reference group.

**Parameter**	**Age group (Years)**		
	**0.5 – 1.5**	**1.5 – 3.0**	**3.0 – 5.0**
*IgM*	*p* < 0.001^a^^−^	*p* = 0.019^a^^−^	*p* < 0.001^a^^−^
	*p* = 0.036^c^^−^	*p* = 0.029^b^^−^	*p* = 0.021^b^^−^
			*p* = 0.002^c−^
IgG total	*p* < 0.000^a^^−^	*p* = 0.031^b^^−^	*p* = 0.003^a^^−^
		*p* = 0.05^c^^−^	*p* = 0.027^c^^−^
IgG1	*p* = 0.016^a^^−^	Δ-	Δ-
IgG2	*p* = 0.045^b^^+^	*p* = 0.049^b^^+^	Δ-/d^+^
		***p*** **= 0.075^d^**^+^	
IgG3	*p* < 0.001^a^^−^	***p*** **= 0.062**^b^^−^	*p* = 0.002^a^^−^
	*p* = 0.048^c^^−^		*p* = 0.022^b^^−^
			*p* = 0.004^c^^−^
IgG4	*p* < 0.001^a^^−^	Δ-	Δ-
	*p* = 0.004^c^^−^		

*Statistical test, Generalized linear model.*

a *= asymptomatic malaria infection.*

b *= sick with malaria infection.*

c *= sick with malaria infection and history of fever in the past 24 h.*

d *= sick without malaria infection.*

− *= reduction.*

+ *= increase.*

Δ *= insignificant change (p ≥ 0.10).*

Δ-/d+ *= variable insignificant change with a change in sick without malaria increasing.*

*NB, Bolded values indicates borderline significant p-values*.

## Discussion

The interesting finding in this study is the higher levels of IgG2 in zinc-deficient subjects contrary to other IgG subclasses and that it was associated with children reported sick with malaria and those sick without malaria in at least all age classes within under-fives. This could mean that IgG2 is more profoundly associated with sickness than protection against malaria.

In *Plasmodium falciparum* malaria, protective immunity to clinical disease is mainly ascribed to immunoglobulin G subclasses. In human malaria, cytophilic IgG1 and IgG3 subclasses with high affinity to Fc receptors on monocytes provide crucial protection (1, 2, 20), gradually increasing with age, to target antigens and the duration of exposure (8). Their protective potential solely rests on their ability in complement fixing, and facilitating opsonization and phagocytosis that limits parasite multiplication in an antibody-dependent manner. The kinetics of antibody isotype formation are continually altered by reinfection (61) with the proportion of cytophilic IgG1 and IgG3 relative to non-cytophilic IgG2 and IgG4 considered more significant than the overall levels of antibodies in providing protection to severe disease (1, 19, 21). It was hypothesized that micronutrient deficiency, zinc deficiency in particular, contributes to alterations in the levels of these antibodies thus influencing protection against malaria since in African children the two entities commonly co-exist. This study provides the first findings associating a specific micronutrient (zinc) deficiency with specific IgG subclasses that confer protection to malaria in endemic areas.

### Variation in Plasma Total IgM and IgG Levels

This study used whole asexual blood stage malaria antigens to reflect the real *in vivo* milieu and provided broader antigenic targets for optimal induction of malaria-specific antibody profiles (15). Primary responses to infection were assessed by measuring plasma levels of IgM and total IgG. The levels changed with malaria status and significant changes were associated with asymptomatic malaria, sick children with malaria infection, and those reported sick with malaria infection and a report of fever in the past 24 h especially in the zinc-deficient group. The similar pattern of change in IgM and total IgG levels support previous reports (2) of switching of IgM on B cells to different isotypes and different IgG subclasses upon encounters with malaria antigens. This implies that IgM primarily determines the magnitude of protection by specific antibodies to malaria pathogens. In addition, Dodoo et al. (62) reported IgM to confer protection and reduce incidence of malaria in African children. The levels of total IgG particularly reacting to glutamate rich protein (GLURP) of the parasite antigenic surface have been reported to be strongly associated with reduced malaria incidence in Africa (63). The significant changes in the zinc-deficient group could be a reflection of persistent infection and concurrent new infections.

### Variation in Plasma Levels of IgG Subclasses

This study found significant changes in all IgG subclasses in the zinc-replete group especially in asymptomatic children and those reported sick with malaria infection (Figure 1). The change in levels for sick children with malaria infection and a report of fever in the past 24 h was high only for IgG3 and IgG4. The predominantly significant change in IgG3 observed in this study concurs with previous studies for the significant role of IgG3 in conferring protection to malaria (1, 9, 14, 16) and the trend that IgG1 and IgG2 are lower than IgG3 and IgG4, respectively, in asymptomatic children is intriguing (Figure 1). It may reflect the potential response that, in asymptomatic malaria, there is a shifting order of response dominance from IgG1 to IgG3 and IgG2 to IgG4, respectively, to sustain protection; albeit studies need to be done to uncover this proposition. The dynamics of this isotype dominance and switching may be due to disease dynamics in the area, with infection and re-infection playing a role in keeping the respective IgG subclass levels high (61).

Higher IgG4 levels in children reported sick with malaria and a report of fever in the previous 24 h may be explained by, firstly, a long half-life of IgG4 (probably due to previous subclinical malaria infection) and, secondly, the report that IgG4 is more associated with disease than protection (64). However, previous study (63) reported IgG3 and IgG4 to be associated with reduced risk of clinical malaria in African children which could also be the case for these findings. In zinc deficiency, however, the higher incremental change of IgG3 in all malaria situations further endorses the significant role of IgG3 in conferring protection against malaria and that probably under deficiencies, the role of this IgG subclass becomes more critical. The findings also contrast previous propositions of downstream isotype switching from IgG3 to IgG1 later in the course of infection after displaying early comparable levels (2). This is because in an area of intense malaria transmission, re-infection maintains the level of IgG3 isotype (17). The significant change in levels of IgG2 in children reported sick with malaria may be associated with disease by the explanation that it probably replaces IgG4 in such situations of micronutrient deficiencies. Generally, there is a clear difference in the alteration of IgG subclass plasma levels in the zinc-deficient group which may be explained by the probable influence of zinc status. IgG subclasses react to various sets of asexual-stage parasite antigenic proteins that may account for the variable effects of zinc deficiency on these antibodies. The consequence of which is an immunological imbalance that largely determines the fate of infection (23) and could have been due to the influence of nutritional status and other factors said to have an impact on the acquisition of natural humoral immunity to *P. falciparum* in endemic areas (22).

### Comparison of Levels of Plasma IgG Subclasses With Respect to Zinc Status

Having assessed the unit change and levels of primary response by IgM and total IgG plasma levels and the unit change in IgG subclasses, the levels of individual IgG subclasses with regard to zinc status were compared (Figure 2). Results showed higher levels of increase in the zinc-replete group as compared to the zinc-deficient group for at least each immunoglobulin subclass. This signifies the importance of IgG subclass coexistence in conferring protection to malaria and that may be influenced by zinc status due to relatively lower increases in the deficient group. While the increase in IgG3 and IgG4 seemed to predominate in the zinc-replete group, IgG3 and partly IgG2 were predominant in the zinc-deficient group. The trend of response for IgG1 and IgG2 and that for IgG3 and IgG4 were also similar in the zinc-replete group but not in the zinc-deficient group. Available reports (63) indicate that, in the course of conferring protection to malaria, the IgG subclasses target different surface parasite antigenic proteins, with IgG3 and IgG4 responding against (glutamate rich protein (GLURP) and IgG1 against apical membrane protein 1 (AMA1). In addition, cytophilic IgG1 and IgG3 have been shown to be differentially regulated over time (59) with IgG3 remaining abundant (56). Such regulation could further explain the association between IgG3 and IgG4 in the zinc-replete group that was lost in the zinc-deficient group. The findings are concordant with previous reports that the proportion of IgG subclasses is more important than their levels in conferring protection against the disease (1, 64, 65). In this study, the proportion seemed to be disturbed under conditions of zinc deficiency. The increase in IgG3 levels can also be explained by its short half-life. IgG3 disappears fast (serum half-life of 7 days) so that a high amount cannot be measured in healthy controls, but there may be many IgG3-producing B cells which rapidly start to produce IgG3 following infection.

In the zinc-deficient group, the predominance of IgG3 and that of IgG2 at least in children reported sick with malaria infection, reflect that probably under zinc deficiency these IgG subclasses are critical in providing protection against the disease. Garraud et al. (66) reported the association between IgG2 and IgG3 in conferring protection to malaria and that certain individuals possess rare mutated alleles encoding an FcR that can bind IgG2 along with IgG3 and IgG1. Other mechanisms have also been suggested (64, 67). Another possibility is that an isotype imbalance (64) occurs in zinc deficiency and thus the resulting IgG2 and IgG3 proportions may not be protective. These results are in contrast to previous findings by Groux and Gysin (68) who found that IgG1 and IgG3 were always predominant in serum. It may be that, in malaria infection as reported previously, either of the cytophilic IgG subclasses (IgG1 or IgG3) should be associated with a non-cytophilic (IgG2 or IgG4) subclass for perfect protection. Kinyanjui et al. (61) reported that the half-lives of these IgG subclasses were probably shorter than what is known (2) with the half-life of IgG3 being shortest of all. The prevailing high levels of IgG3 in both zinc-replete and zinc-deficient groups may be attributed to continued exposure to low but persistent malaria infections in an endemic area (1). Rzepczyk *et al*. (17) reported skewing of IgG response toward a short-lived IgG3 in response to *P. falciparum* infection and that plasma levels could be maintained through persistent infection or new infection.

### Association Between IgG3 and IgG4 Subclasses Under Different Conditions of Micronutrients and Malaria Status

Evaluation on the potential impact of zinc deficiency, magnesium deficiency, iron deficiency anemia, or malaria status on the association between IgG3 and IgG4 plasma level yielded no evidence (Figure 3). Such findings imply that deficiency in micronutrients might influence production and consequently proportions of protective antibodies to malaria but not their association.

### Effects of Zinc Deficiency: Does Age Make a Difference?

Several reports have evaluated the profile of antibody response to asexual blood stages of the malaria parasite. Some have, in principle, proposed the gradual change in the naturally acquired antibody responses with age (1, 2, 9, 43). This study adds more information on zinc deficiency variably influencing the profile of antibody protection to malaria. Zinc deficiency seemed to significantly and negatively influence the profile of IgM and IgG in all age groups implying that the primary response is generally influenced by zinc deficiency regardless of age differences (Table 3). Children of 0.5 – 1.5 years have shown to be prone to lowered IgG1 and IgG4 production in zinc deficiency while the impact on IgG2 and IgG3 is significant in all age groups with IgG2 increasing with zinc deficiency in contrast with IgG3. As suggested by previous studies, IgG3 levels seem to be prevalent in all age groups implying its critical role in conferring protection across all young ages (within <5 years). Deficiency in zinc may be more alarming in endemic areas due to its impact on the largely believed immunoprotective IgG3 subclass and possibly other IgG subclasses that have been shown in this study. Lower levels may occur with any other micronutrient deficiency, zinc deficiency particularly, and could be a result of deficiency in a cohort of dietary nutrients whose effect could be on a number of host memory CD4 T cells generated in the course of infection. Consequently, a decay in immunity can occur (69), leading to immune dysfunction (40), and generally impact natural humoral immunity (22). Immunological balance is key if the host is to win against malaria infection and thus is key for the fate of infection (23). This balance is largely influenced by nutritional status necessitating the need for proper medical-nutritional management (48) to prevent adverse effects of malaria infection (49) and thus result in a good outcome in treatment. Nevertheless exposure to malaria might be critical in determining variations in antibody responses to malaria, with those with previous and frequent periodic exposures throughout their lives being subject to higher antibody titers than unexposed and less frequently infected individuals (51, 52).

## Conclusion

These findings have shown, preliminarily, the variable effects that zinc may have on the profiles of IgM, IgG total, and IgG subclasses, and that these effects seem to vary with age. IgG3 has been shown to be critically affected across all age classes as per this study's age classification. Inclusion of appropriately selected micronutrients could be a way forward toward boosting the production of protective IgG subclasses in endemic areas. Previously we found a profound impact of zinc and other micronutrients on the cytokine arm of immune responses, both in the innate and anti-inflammatory cytokine profiles (54, 55). This evidence enables us to firmly recommend the inclusion of micronutrients in future malaria vaccine programs pending further thorough and extensive studies on these interactions. Special attention should be paid to isotype switching under conditions of micronutrient deficiencies, malaria status, and age.

### Limitations of the Study

The initial plan was to conduct this study in three districts with the highest malaria prevalence and potential micronutrient deficiencies in under-fives. These included Handeni, Muheza, and Korogwe. Finally we ended up basing in Handeni district only as it was not possible to carry out this study in the other two districts due to the existence of other malaria projects. Thus children missed the opportunity to participate in this study.

### Strengths of the Study

The study developed its own ELISA protocol that produced results reported in this paper but also created the basis for the bigger randomized controlled micronutrient supplementation trial that followed afterward. Socially, we built a health facility in Bondo village for the project that currently is used as a health facility.

## Data Availability Statement

The raw data supporting the conclusions of this article will be made available by the authors, without undue reservation.

## Ethics Statement

The studies involving human participants were reviewed and approved by the National Institute for Medical Research Ethics Sub-committee. Written informed consent to participate in this study was provided by the participants' legal guardian/next of kin.

## Author Contributions

EM developed the protocol, participated in laboratory and data analysis, drafting and writing the manuscript, and critically reviewed the final version for submission to the publishing journal. GH, JV, HV, and HS participated in protocol development, design, field work, conducted laboratory, and data analysis. JC participated in drafting the manuscript and critical review before submission. In addition, HV and HS conceived the study, acquired funds, directed the field work, co-supervised data analysis and manuscript preparation, and critically reviewed and approved of final version for submission. All authors approved the final version for submission to the journal for publication.

## Funding

This study received financial support from the Netherlands Organization for Scientific Research, NWO/WOTRO (grant numbers W93-413, WAO93-441, and WIZ93-465) and UN Children's Fund (UNICEF).

## Conflict of Interest

The authors declare that the research was conducted in the absence of any commercial or financial relationships that could be construed as a potential conflict of interest.

## Publisher's Note

All claims expressed in this article are solely those of the authors and do not necessarily represent those of their affiliated organizations, or those of the publisher, the editors and the reviewers. Any product that may be evaluated in this article, or claim that may be made by its manufacturer, is not guaranteed or endorsed by the publisher.
